# Successful Excision of Gynecomastia with Nipple Repositioning Technique Utilizing the Dermoglandular Flap

**Published:** 2015-07

**Authors:** Sadrollah Motamed, Seyed Esmail Hassanpour, Seyed Mehdi Moosavizadeh, Ataollah Heidari, Abdoreza Rouientan, Mahmood Nazemian

**Affiliations:** 1 Department of Plastic and Reconstructive Surgery, 15 Khordad Hospital, Shahid Beheshti University of Medical Sciences, Tehran, Iran;; 2 Department of Plastic and Reconstructive Surgery, Shahid Modares Hospital, Shahid Beheshti University of Medical Sciences, Tehran, Iran

**Keywords:** Gynecomastia, Nipple repositioning, Dermoglandular flap

## Abstract

There are many surgical techniques for treating gynecomastia. We report a new surgical technique in an adolescent with fatty glandular gynecomastia grade III, who was referred from an endocrinologist to our clinic. We excised the gynecomastia with nipple repositioning utilizing the dermoglandular flap (about 1 cm thickness and 10 cm width). After one month, no complication was detected and the patient was satisfied with his new breasts. We suggest this technique for fatty glandular gynecomastia grade III.

## INTRODUCTION

Testosterone replacement therapy (TRT) in hypogonadal men has become more common, as health care providers have become increasingly aware of the benefits of treatment. However, administration of exogenous testosterone can be associated with side effects on the prostate, fertility and development of gynecomastia.^1^ Gynecomastia is believed to arise from peripheral conversion of testosterone to estradiol via the enzyme aromatase. The condition is embarrassing to men, and may cause discontinuation of testosterone treatment that has otherwise been successful. Some medical treatments for this problem are recommended, but, sometimes they can not resolve this problem and endocrinologist refer these patients to plastic surgeon. 

In this situation, the indication for surgical treatment of gynecomastia are based on two main objectives including (i) The restoration of male chest shape and countering; and (ii) Diagnostic evaluation of suspected breast lesions.^2^ The diagnostic evaluation begins with an adequate history and a thorough breast examination helped by laboratory tests and instrumental research. There are many different classifications of gynecomastia. Such as Webster classified gynecomastia consisted of (i) Glandular, (ii) Fatty glandular and (iii) Simple fatty categories.

Hypertrophy of the stromal and glandular breast tissue is the most common type of gynecomastia seen among adolescents. The classification in 1973 is based on the amount of breast enlargement (grade I to III). Here, we report a new surgical technique in a hypogonadal young man with glandular dominant, fatty glandular gynecomastia due to testosterone while his inframammary fold (IMF) and nipple-areola complex (NAC) were below ideal position, and he had lateral chest wall roll (grade III).^3^ Six months after this operation, he was satisfied with his new IMF, NAC and breasts contour while no complication was detected. 

## CASE REPORT

A married man aged 24 years was referred in 2014 for gynecomastia to the clinic affiliated to Department of Plastic and Reconstructive Surgery, Shahid Modares Hospital, Shahid Beheshti University of Medical Sciences, Tehran, Iran. His medical history denoted to an initiation in 2011. He was treated for bilateral undescended testis (UDT). According to operation notes, laparoscopy was performed and right orchiectomy and left orchieopexy were done for him. Azoospermia was confirmed for him in 2012. Because of delayed puberty, loss of body and facial hair, azoospermia and etc., the patient referred to an endocrinology clinic and his laboratory results showed: TSH=1.5 µ Iu/ml, FSH=58.6 Iu/l, LH=22.6 Iu/l, prolactin=28.3 ng/ml, testosterone=0.4 ng/ml while the liver function test (LFT) and beta HCG and alpha-fetoprotein (AFP) were in normal range. His karyotype finding was 46 and XY was compatible with an apparently normal male from cytogenetic point of view.

**Fig. 1:**
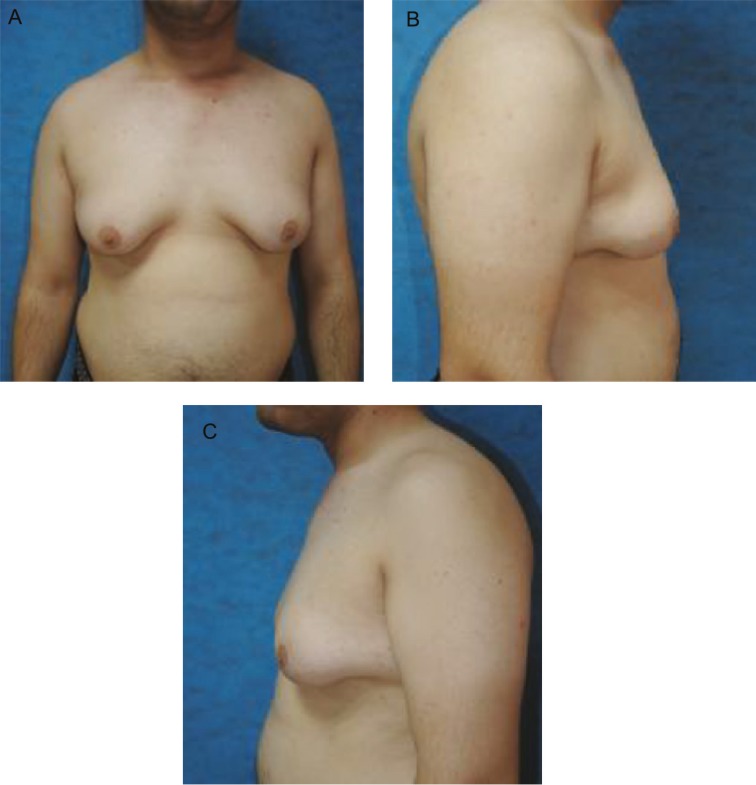
**A)** Preoperative view (AP).** B)** Preoperative view (right).**C)** Preoperative view (left).

Scrotal sonography showed a small and atrophic (12×6 mm) left testis denoted to a diffuse decreased echo and the right testis was not detectable. The patient was treated with testosterone (250 mg every 3 weeks) and was closely followed in endocrinology clinic every 3 months. After one year, testosterone level reached 11.9 ng/ml with increased facial and body hair and libido, and his mild gynecomostia status progressed to a severe type. Breast sonography showed severe glandular dominant, fatty glandular gynecomastia. Sperm analysis revealed azoospermia. Testostrone was continued for him and was referred to an infertility clinic for search of a donor sperm and to a plastic surgery clinic due to gynecomastia (Figure 1A-C).

**Fig. 2:**
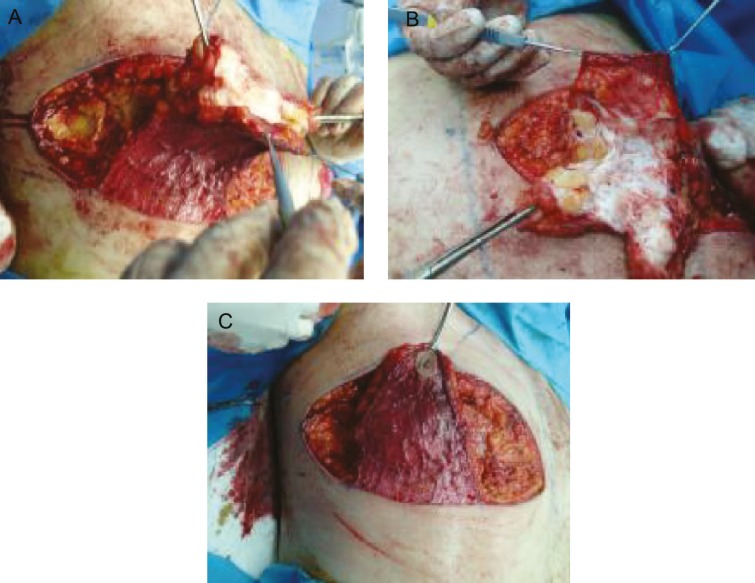
**A) **Intra operative (During dissection of pedicle). **B) **Intra operative (Narrowed pedicle). **C) **Intraoperative view.

**Fig. 3:**
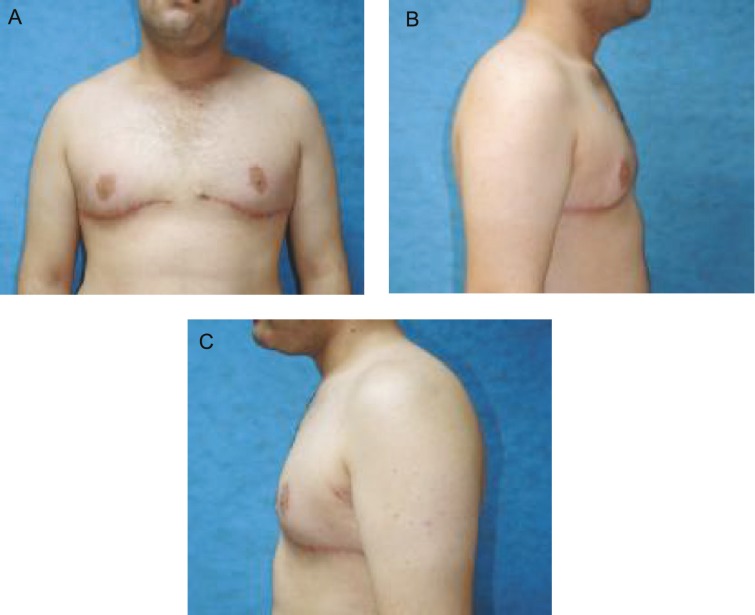
**A) **Postoperative view (AP). **B) **Postoperative view (right). **C) **Postoperative view (left).

On examination, NAC and IMF were below ideal position, a significant lateral chest wall roll was present, and upper abdominal laxity and high skin redundancy were detectable. We choose a new technique with good aesthetic result, involved elevation of the nipple areola complex on a thin dermoglandular flap (1 cm diameter, 10 cm width), which was transposed under a superior chest wall flap (Figure 2A-C). Excision of the soft tissue under the flap thinned the chest. Aggressive breast volume reduction, nipple repositioning, good scar position (around the areola and at the new infra-mammary fold) were possible. Lateral extension of the chest excision dealt with the lateral chest roll (Figure 3A-C).

The patient was visited the first day, week and month after surgery. No seroma, necrosis, asymetry, malposition, fever, discoloration were detected. Sensation was normal and the patient was satisfied with his new IMF, NAC, and breasts contours. His preoperative, intra-operative after the first day, first week and first month post-operative were shown in Figures 1-3.

## DISCUSSION

Numerous techniques have been advocated to treat gynecomastia with many procedures leaving a periareolar scar. In the past two decades, there has been a shift from the open approach to minimally invasive techniques while the current literature supports the use of liposuction/ultrasonnd–assisted liposuction alone or in combination with driver excision of the residual breast tissue using either a periareolar incision or a pall–through technique with a remote incision–although liposuction alone is ideal for gynecomstia with a predominantly fatty breast. It leaves a remnant behind the nipple–arcola complex in patients who have fibrous/glandular hyperplasia.^4^

Liposuction through a periareolar incision combined with removal of residual breast tissue (through this same incision) has several drawbacks. Besides, there is shearing and trauma to the nipple and the possibility of a thermal burn at this site. It also combines, namely, liposuction and open excision.^5^ Excisional techniques in patients with significant excess skin and grade III ptosis are still necessary for patients with a glandular component benefit from direct glandular excision (subcutaneous mastectomy) as liposuction will not greatly affect firm, subareolar tissue.^1^^,^^4^

A preriareolar incision, keeping the scar in an aesthetic position provides access to the gland. Complication secondary to subcutaneous mastectomy include changes to nipple sensation, breast asymmetry hypertrophic or keloid scarring and nipple necrosis.^1^^,^^4^ For patients with moderate to severe skin and soft tissue excess requiring nipple elevation, liposuction alone is not adequate. Excisional procedures that remove skin and soft tissue, while resizing and repositioning the nipple areola are required.^5^^,^^6^

We used a technique with good aesthetic results by elevation of the nipple areola complex on a thin dermoglandular flap which was transposed under a superior chest wall flap. Excision of the soft tissues under the flap thins the chest. Aggressive breast volume reduction, nipple repositioning, good scar position (around the areola and at the new IMF) was possible. Lateral extension of the chest excision deals with commonly found lateral chest roll. The treatment of choice for patients requiring excision and nipple areola repositioning is an elliptical excision based on the current IMF with internal extension to treat lateral thoracic rolls.^3^^,^^5^^-^^7^

Selection of surgical technique must take into account for the relative excesses of skin, fat and glandular tissue, nipple position, patient and surgeon preference, as well as the willingness of the patient to accept surgical scars in exchange for better chest contour in more severe cases.^6^

According to this discussion and rely to our patient who had grad III gynecomastia, large size and presence of excess skin and predominance of glandular tissue, it was difficult to achieve good aesthetic outcome with liposuction alone or free nipple graft. Thereby, we preferred modified reduction mammoplasty with a dermal pedicle as excisional gynecomastia with elevation of the nipple areola complex on a dermoglandular pedicle (on a thin inferiorly based dermoglandular pedicle). 

Using this technique, we found good results regarding a good symmetry, good contouring, size, shape, and NAC position with complete satisfaction of the patient. Although the patient would undergo an IMF incision, the function and psychological sequlae of a grade III breast associated with ptosis would be relieved with this technique. Whit this approach, secondary procedures may rarely be required.

## CONFLICT OF INTEREST

The authors declare no conflict of interest.

## Note

Please cite this paper as:

Motamed S, Hassanpour SE, Moosavizadeh SM, Heidari A, Rouientan A, Nazemian M. Successful Excision of Gynecomastia with Nipple Repositioning Technique Utilizing the Dermoglandular Flap. World J Plast Surg 2015;4(2):163-167.
